# Late hypo-attenuated annular narrowing 1 year after transcatheter aortic valve implantation with a Navitor valve

**DOI:** 10.1093/ehjcr/ytae224

**Published:** 2024-05-02

**Authors:** Juri Iwata, Kentaro Hayashida, Hideyuki Shimizu, Masaki Ieda

**Affiliations:** Department of Cardiology, Keio University School of Medicine, 35 Shinanomachi, Shinjuku-ku, Tokyo 160-8582, Japan; Department of Cardiology, Keio University School of Medicine, 35 Shinanomachi, Shinjuku-ku, Tokyo 160-8582, Japan; Department of Cardiovascular Surgery, Keio University School of Medicine, Tokyo, Japan; Department of Cardiology, Keio University School of Medicine, 35 Shinanomachi, Shinjuku-ku, Tokyo 160-8582, Japan

## Case description

A 91-year-old female patient diagnosed with very severe aortic stenosis (AS) underwent transcatheter aortic valve implantation (TAVI) with a 23 mm Navitor valve. Preoperative multidetector computed tomography (MDCT) demonstrated an annular area of 303 mm^2^, with calcification in the left ventricular outflow tract.

**Figure 1:**
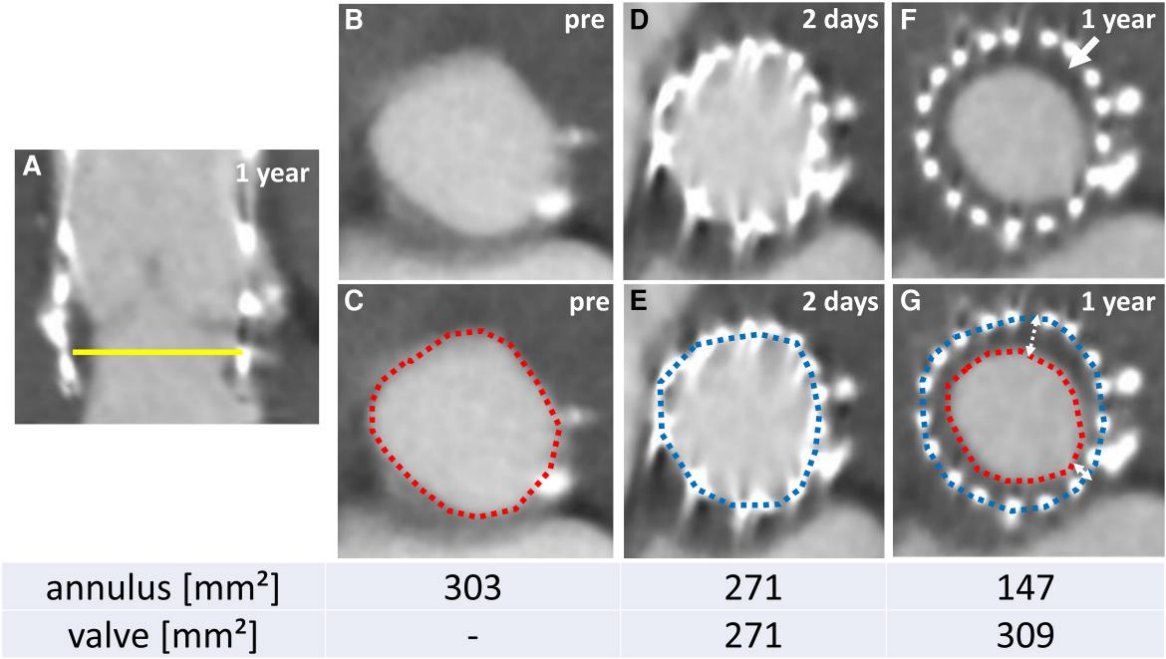
Computed tomography measurements. (*A*) Long-axis 1 year after transcatheter aortic valve implantation. (*B*–*G*) Short axis at the height of the yellow line. (*B*, *C*) Preoperative. (*D*, *E*) Two days after transcatheter aortic valve implantation. (*F*, *G*) One year after transcatheter aortic valve implantation. Red and blue lines indicate annulus and valve sizes, respectively; the white arrow in (*F*) indicates hypo-attenuated annular narrowing; the dotted double arrow in (*G*) indicates the distance between the valve and annulus.

After TAVI, the patient on clopidogrel 75 mg was followed by MDCT 2 days and 1 year postoperatively. Multidetector computed tomography at 1 year demonstrated ∼50% reduction in the annular area (*Figure 1C* and *G*: red line, from 303 to 147 mm^2^) with ‘hypo-attenuated annular narrowing’ (HAN) inside the Navitor valve (*Figure 1F*, arrows). Conversely, the Navitor stent was slightly expanded (*Figure 1E* and *G*: blue line, from 271 to 309 mm^2^, ∼10% in area). The HAN was less prominent in the 4 o’clock direction of the annulus than in the 12 o’clock direction (*Figure 1G*, dotted double arrow). Transthoracic echocardiography revealed no increase in the mean pressure gradient (5 mmHg) or para/transvalvular regurgitation over 1 year.

The potential mechanism underlying this finding could be pannus, thrombus formation, or normal endothelization. Metal artefacts in the stent flame make it difficult to obtain precise measurements of MDCT value. However, annular narrowing was less prominent around calcification. Therefore, HAN could be a ‘pannus formation’ caused by direct contact and continuous compression by the valve to the annulus. Despite a 50% loss in the annular area, no impact was observed on the haemodynamics with HAN, owing to the superior haemodynamic performance of the Navitor.

Few reports have described pannus formation after TAVI.^1^ However, we are the first to report significant HAN 1 year after TAVI with a Navitor. This phenomenon was more frequently observed with Navitor than with Evolut series in our hospital (unpublished data).

Elucidation of the mechanism and its clinical impact, and identification of the predictors of this finding in a larger cohort would be valuable.

**Consent:** The study participant provided informed consent for the use of data before the procedure.

**Funding:** None declared.

## Data Availability

The data underlying this article will be shared upon reasonable request to the corresponding author.
